# Sex-specific risk factor profile in oesophageal adenocarcinoma

**DOI:** 10.1038/sj.bjc.6604701

**Published:** 2008-10-07

**Authors:** H E Löfdahl, Y Lu, J Lagergren

**Affiliations:** 1 Unit of Esophageal and Gastric Research (ESOGAR), Department of Molecular Medicine and Surgery, Karolinska Institutet, Stockholm, Sweden; 2 Department of Medical Epidemiology and Biostatistics, Karolinska Institutet, Stockholm, Sweden

**Keywords:** gender, neoplasm, oesophagus, cardia, population based

## Abstract

A nationwide Swedish case–control study of 388 men and 63 women with adenocarcinoma of the oesophagus and gastro-oesophageal function and 676 men controls and 140 women investigated whether sex differences in aetiology contribute to male predominance. Compared with men, women seemed more vulnerable to reflux (odds ratio (OR)=4.6, 95% confidence interval (CI)=2.0–10.5 *vs* OR=3.4, 95% CI=2.5–4.6), obesity (OR=10.3, 95% CI=2.6–42.3 *vs* OR=5.4, 95% CI=2.6–10.8) and smoking (OR=5.3, 95% CI=2.0–14.1 *vs* OR=2.8, 95% CI=1.9–4.2), less harmed by low intake of fruit and vegetables (OR=0.9, 95% CI 0.3–2.4 *vs* OR=1.6, 95% CI=1.1–2.2) and less protected by *Helicobacter pylori* infection (OR=0.5, 95% CI=0.3–0.8 *vs* OR=1.6, 95% CI=0.5–5.4).

The striking 6–8: 1 male predominance of adenocarcinoma of the oesophagus, including the gastro-oesophageal junction (Vizcaino *et al*, 2002), has not yet been explained. An understanding of this gender difference might help explain why the incidence of this tumour is increasing more rapidly than that of any other malignancy in several populations (Vizcaino *et al*, 2002), and may help to identify preventive and therapeutic strategies. Three established risk factors are gastro-oesophageal reflux (Lagergren *et al*, 1999a; Shaheen and Ransohoff, 2002; Vakil *et al*, 2006), obesity (Lagergren *et al*, 1999b) and tobacco smoking (Lagergren *et al*, 2000), whereas the two protective factors are high intake of fruits and vegetables (Terry *et al*, 2001) and infection with *Helicobacter pylori (H. pylori)* (Ye *et al*, 2004; Rokkas *et al*, 2007). We hypothesised that male predominance in oesophageal adenocarcinoma is due atleast to established risk factors having a more harmful effect on men than women and that protective factors have a stronger preventive effect on women than men. To test this hypothesis, we conducted gender-specific analyses of the five established aetiologic factors in a population-based case–control study in Sweden.

## Methods

We have previously examined the five established aetiological factors in our Swedish population-based case–control study of adenocarcinoma of the oesophagus and gastro-oesophageal junction (Lagergren *et al*, 1999a, 1999b, 2000; Terry *et al*, 2001; Ye *et al*, 2004), but gender-specific analyses or analyses combining both these sites have not been presented. The study design is described elsewhere (Lagergren *et al*, 1999a). Briefly, it is based on the entire Swedish-born population, living in Sweden during 1995–97, below 80 years of age. Cases were identified shortly after diagnosis through collaboration among all 195 relevant hospital departments and all six tumour registers, which reduced non-participation due to weakness or early mortality. To decrease misclassification, special routines for documentation and prospective reporting of tumour site and type were used by endoscopists, surgeons and pathologists. The tumour had to be adenocarcinoma in type and to have its centre within the oesophagus or a maximum 3 cm distal to the gastro-oesophageal junction. The tumour specimens were finally re-examined by one pathologist. Control participants were randomly selected from the population register and frequency-matched to cases for age and sex. Participants were interviewed face-to-face about exposures and background data by professional interviewers who were unaware of the study hypotheses and trained to deal with the cases and controls in exactly the same manner. Regarding reflux, questions were asked about recurrent heartburn and regurgitation, that is, the symptoms on which a diagnosis of gastro-oesophageal reflux is based (Vakil *et al*, 2006). To avoid tumour influence on the reported exposure, we disregarded reflux symptoms less than 5 years before the interview. To assess the effects of body mass index (BMI), participants were asked about their weight and height 20 years before the interview, their maximum and minimum weight as adults, and their weight at the age of 20 years (Lagergren *et al*, 1999b). Concerning tobacco smoking, the user status was recorded as any type of tobacco use 2 years before the interview, whereas frequency and duration of tobacco smoking was based on cigarette smoking alone (Lagergren *et al*, 2000). The total average consumption of all fruits and vegetables 20 years before the interview was assessed from a food frequency questionnaire (Terry *et al*, 2001). The participants were asked to provide a venous blood sample for *H. pylori* testing. Serum immunoglobulin G antibodies against *H. pylori* and its virulence factor Cag A were measured by immunosorbent assay (ELISA) as described earlier (Ye *et al*, 2004).

Unconditional logistic regression was used to estimate odds ratios (ORs) with 95% confidence intervals (CIs) for all study exposures in sex-stratified analyses. In multivariable models, the ORs were adjusted for age, educational level, alcohol use, reflux, BMI, tobacco smoking, intake of fruits and vegetables, and *H. pylori* infection. The categorisation of each of these included variables in the model is presented in Tables 1 and 2.

## Results

Among 529 eligible cases and 1128 eligible controls, 451 (85%) and 816 (72%), respectively, participated in the interview. Among the cases, 63 were women (14%), with a male-to-female ratio of 7 : 1. Some characteristics of the study participants are presented in Table 1. Most patients were aged 70–79 years. Female cases had on average, a shorter education than controls and male cases and controls. Use of alcohol was higher among men, but similar between cases and controls in each sex. Table 2 presents an overview of the sex-stratified risk estimates of the five studied aetiological factors in relation to risk of oesophageal and junctional adenocarcinoma. Reflux symptoms showed a slightly higher risk estimate in women (OR 4.6, 95% CI 2.0–10.5) than in men (OR 3.4, 95% CI 2.5–4.6) at least weekly. An increased severity and duration of reflux was associated with higher risk estimates among women than among men (Table 3).

Women with a long history of reflux (>20 years) had an OR of 12.3 (95% CI 2.9–51.4), whereas the corresponding OR in men was 6.6 (95% CI 3.8–11.6). A high BMI was associated with higher point estimates in women than in men (Tables 2 and 4); obese women had an OR of 10.3 (95% CI 2.6–42.3) and obese men of 5.4 (95% CI 2.6–10.8). Also, a greater increase in BMI between the lowest and highest adult BMI rendered a higher OR among women than among men (Table 4). Current tobacco smoking was associated with a higher risk estimate in women (OR 5.3, 95% CI 2.0–14.1) than in men (OR 2.8, 95% CI 1.9–4.2), and a higher frequency of cigarette smoking (>19 per day) showed a higher risk in women (OR 5.7, 95% CI 1.3–25.6) than in men (OR 2.3, 95% CI 1.6–3.4) (Table 5). An increased risk associated with low intake of fruits and vegetables was found only in men (men OR 1.6, 95% CI 1.1–2.2; women 0.9, 95% CI 0.3–2.4) (Table 2). The inverse risk among *H. pylori*-infected men (OR 0.5, 95% CI 0.3–0.8) was not seen in women (OR 1.6, 95% CI 0.5–5.4) (Table 2). The point estimates from separate analyses of oesophageal and gastro-oesophageal junctional adenocarcinoma did not reveal any material male–female differences (data not shown).

## Discussion

This study finds, in contradiction to our hypothesis, that exposures to reflux, obesity and tobacco are stronger risk factors for oesophageal adenocarcinoma in women than in men and that the protective effects of fruits and vegetables and infection with *H. pylori* observed in men do not occur in women.

Study strengths include the nationwide and population-based design with high participation, face-to-face interviews, detailed exposure information and a thorough tumour classification. Although we included virtually all female cases in the whole of Sweden during a 3-year period, to offset the low incidence of oesophageal adenocarcinoma in women, we combined it with adenocarcinoma of gastro-oesophageal junction, as justified by their similar sex distributions and risk factor profiles (Vizcaino *et al*, 2002).

During the last few years, it has been established that reflux and obesity (Lagergren *et al*, 1999a, 1999b; Shaheen and Ransohoff, 2002; Vakil *et al*, 2006) are independent and strong risk factors for oesophageal adenocarcinoma, whereas tobacco smoking is a moderate risk factor (Lagergren *et al*, 2000). Dietary intake of fruits and vegetables (Terry *et al*, 2001) and infection with *H. pylori* (Ye *et al*, 2004; Rokkas *et al*, 2007) are inversely associated with risk of this cancer. All these results are entirely in agreement with the findings in our case–control study (Lagergren *et al*, 1999a, 1999b, 2000; Terry *et al*, 2001; Ye *et al*, 2004), indicating good validity. As however, a striking majority of the patients are men and most studies have not analysed women separately, available work has established aetiologic factors only among men. This study, undertaken mainly to compare risk factor profiles among women and men using a large data source of high validity, finds that women do not seem less vulnerable to risk exposures or more protected by preventive factors than men, but rather suggests the opposite. A possible explanation might be that women are exclusively protected from developing oesophageal adenocarcinoma by some yet unidentified factors, for example, female sex hormones. A protective role of oestrogen has been evaluated in some studies, but with no clear evidence of the effect, although the statistical power was limited (Lagergren and Jansson, 2005; Chandanos *et al*, 2006). However, a UK case–control study noted a decreased risk among women with a history of breast feeding (Cheng *et al*, 2000).

This study suggests that with regard to oesophageal adenocarcinoma risk, women are as vulnerable as men to exposure to reflux, obesity and tobacco, whereas the protective effects of fruits and vegetables and infection with *H. pylori* might be confined to men. Thus, the sex difference in oesophageal adenocarcinoma does not seem to be explained by differences in risk factor profiles of known aetiological agents.

## Figures and Tables

**Table 1:** Characteristics of female and male cases and controls

	**Women**		**Men**	
	**Cases**	**Controls**	**Cases**	**Controls**
**Variables**	***N* (%)**	***N* (%)**	***N* (%)**	***N* (%)**
Total	63	140	388	676
				
*Age group (years at interview)*				
0–49	5 (7.9)	10 (7.1)	28 (7.2)	38 (5.6)
50–59	9 (14.3)	24 (17.1)	71 (18.3)	136 (20.0)
60–69	14 (22.2)	39 (27.9)	131 (33.8)	206 (30.5)
70–79	35 (55.6)	67 (47.9)	158 (40.7)	296 (43.8)
				
*Formal education (years)*				
0–9	45 (71.4)	74 (52.9)	266 (68.6)	423 (62.6)
10–12	12 (19.1)	33 (23.6)	68 (17.5)	126 (18.6)
>13	6 (9.5)	33 (23.6)	54 (13.9)	127 (18.8)
				
*Alcohol status (current usage 20 years before interview)*				
No	20 (31.8)	39 (27.9)	55 (14.2)	92 (13.6)
Yes	43 (68.3)	101 (72.1)	333 (85.8)	584 (86.4)

**Table 2:** Risk factor profile, women *vs* men, for oesophageal and cardia adenocarcinoma, expressed in OR with 95% CI

	**Women**			**Men**		
	**Controls**	**Cases**	**OR^a^**	**Controls**	**Cases**	**OR^a^**
**Variables**	***N*=140 (%)**	***N*=63 (%)**	**(95% CI)**	***N*=676 (%)**	***N*=388 (%)**	**(95% CI)**
*Reflux (at least weekly 5 years or more before interview)*						
No	17 (12)	25 (40)	1.0 (reference)	118 (17)	163 (42)	1.0 (reference)
Yes	123 (88)	38 (60)	4.6 (2.0–10.5)	558 (83)	225 (58)	3.4 (2.5–4.6)
						
*BMI (20 years before interview)*						
<22	56 (40)	12 (19)	1.0 (reference)	151 (22)	45 (12)	1.0 (reference)
22.0–24.9	55 (39)	25 (40)	2.4 (0.9–6.0)	311 (46)	143 (37)	1.5 (1.0–2.3)
25.0–29.9	22 (16)	16 (25)	4.3 (1.4–13.1)	196 (29)	164 (42)	2.7 (1.8–4.1)
⩾30.0	7 (5)	10 (16)	10.3 (2.6–42.3)	18 (3)	36 (9)	5.4 (2.6–10.8)
						
*Cigarette-smoking status (2 years before interview)*						
Never	90 (64)	29 (46)	1.0 (reference)	233 (35)	71 (18)	1.0 (reference)
Previous	28 (20)	13 (21)	1.9 (0.7–5.1)	285 (42)	200 (52)	2.3 (1.6–3.3)
Current	22 (16)	21 (33)	5.3 (2.0–14.1)	158 (23)	117 (30)	2.8 (1.9–4.2)
						
*Intake of fruits and vegetables (20 years before interview)*						
Highest	63 (45)	23 (37)	1.0 (reference)	213 (32)	95 (25)	1.0 (reference)
Medium	50 (36)	27 (43)	1.1 (0.5–2.5)	275 (41)	140 (36)	1.0 (0.7–1.4)
Lowest	27 (19)	13 (21)	0.9 (0.3–2.4)	188 (28)	153 (39)	1.6 (1.1–2.2)
						
*Helicobacter pylori (HP) status and its virulence factor CagA status*						
Negative HP and CagA	34 (24)	10 (16)	1.0 (reference)	161 (24)	90 (23)	1.0 (reference)
Mixed HP or CagA	25 (18)	7 (11)	1.3 (0.4–4.7)	92 (13)	68 (18)	1.2 (0.8–1.9)
Positive HP and CagA	26 (19)	10 (16)	1.6 (0.5–5.4)	161 (24)	45 (12)	0.5 (0.3–0.8)
Missing	55 (39)	36 (57)	2.8 (1.0–7.4)	262 (39)	185 (48)	1.1 (0.8–1.5)

BMI=body mass index; CI=confidence interval; OR=odds ratio.

a Adjustments were made for age, educational level, alcohol use, BMI, cigarette smoking, intake of fruits and vegetables, and *Helicobacter pylori* infection.

**Table 3:** Gastro-oesophageal reflux symptoms and risk of oesophageal and cardia adenocarcinoma expressed in OR with 95% CI

	**Women**			**Men**		
	**Controls**	**Cases**	**OR^a^**	**Controls**	**Cases**	**OR^a^**
**Variables**	***N*=140 (%)**	***N*=63 (%)**	**(95% CI)**	***N*=676 (%)**	***N*=388 (%)**	**(95% CI)**
*Reflux at least weekly and/or at night*						
No	123 (88)	38 (60)	1.0 (reference)	558 (83)	225 (58)	1.0 (reference)
Reflux, but not at night	9 (6)	8 (13)	2.2 (0.7–7.3)	60 (9)	47 (12)	1.8 (1.2–2.8)
Reflux at night	8 (6)	17 (27)	7.6 (2.7–21.5)	58 (9)	116 (30)	5.1 (3.5–7.4)
						
*Frequency of reflux symptoms*						
Never	123 (88)	38 (60)	1.0 (reference)	558 (83)	225 (58)	1.0 (reference)
1 per week	12 (7)	9 (14)	1.9 (0.6–5.6)	83 (12)	58 (15)	1.7 (1.2–2.6)
2 or more per week	5 (4)	16 (26)	12.5 (3.7–42.5)	35 (5)	105 (27)	7.2 (4.6–11.3)
						
*Reflux symptom score* b						
No symptoms	123 (88)	38 (60)	1.0 (reference)	558 (83)	225 (58)	1.0 (reference)
1–2 points	8 (6)	5 (8)	1.4 (0.3–5.5)	50 (7)	32 (8)	1.5 (0.9–2.6)
2.5–4 points	5 (4)	7 (11)	4.4 (1.1–17.1)	38 (6)	53 (14)	3.3 (2.0–5.3)
4.5–6.5 points	4 (3)	13 (21)	13.0 (3.4–49.6)	30 (4)	78 (20)	6.7 (4.1–10.9)
						
*Duration of reflux symptoms*						
No symptoms	123 (88)	38 (60)	1.0 (reference)	558 (83)	225 (58)	1.0 (reference)
<12 years	8 (6)	6 (10)	2.0 (0.5–7.7)	33 (5)	44 (11)	3.2 (1.9–5.3)
12–20 years	6 (4)	8 (13)	3.9 (1.1–14.5)	61 (9)	68 (18)	2.4 (1.6–3.7)
>20 years	3 (2)	11 (18)	12.3 (2.9–51.4)	24 (4)	51 (13)	6.6 (3.8–11.6)

BMI=body mass index; CI=confidence interval; OR=odds ratio.

a Adjustments were made for age, educational level, alcohol use, BMI, cigarette smoking, intake of fruits and vegetables, and *Helicobacter pylori* infection.

b The index score included symptom characteristics (heartburn only=1 point, regurgitation only=1 point, heartburn and regurgitation combined=1.5 points), nightly symptoms (no=0 point, yes=2 points) and symptom frequency (once a week=0 point, 2–6 times a week=1 point, 7–15 times a week=2 points, >15 times a week=3 points).

**Table 4:** BMI and risk of oesophageal and cardia adenocarcinoma expressed in OR with 95% CI

	**Women**			**Men**		
	**Controls**	**Cases**	**OR^a^**	**Controls**	**Cases**	**OR^a^**
**Variables**	***N*=140 (%)**	***N*=63 (%)**	**(95% CI)**	***N*=676 (%)**	***N*=388 (%)**	**(95% CI)**
*BMI maximum as adult*						
<22	18 (13)	2 (3)	1.0 (reference)	40 (6)	12 (3)	1.0 (reference)
22–24.9	44 (31)	11 (17)	1.1 (0.2–6.3)	198 (29)	77 (20)	1.1 (0.5–2.4)
25–29.9	56 (40)	26 (41)	3.1 (0.6–16.9)	335 (50)	208 (54)	1.8 (0.9–3.6)
⩾30	22 (16)	24 (38)	6.5 (1.1–39.0)	103 (15)	91 (23)	2.2 (1.1–4.7)
						
*BMI minimum as adult*						
<22	112 (80)	32 (51)	1.0 (reference)	352 (52)	147 (38)	1.0 (reference)
22–24.9	23 (16)	24 (38)	4.1 (1.7–9.6)	270 (40)	183 (47)	1.6 (1.2–2.2)
⩾25	5 (4)	7 (12)	7.6 (1.9–30.5)	54 (8)	58 (15)	2.7(1.7–4.2)
						
*BMI change during adult life*						
>2	15 (11)	4 (6)	1.0 (reference)	121 (18)	59 (15)	1.0 (reference)
2–4.9	51 (36)	22 (35)	1.3 (0.3–5.5)	283 (42)	155 (40)	1.0 (0.6–1.4)
5–8.9	53 (38)	21 (33)	1.0 (0.2–4.0)	214 (32)	128 (33)	1.0 (0.7–1.5)
⩾9	21 (15)	16 (25)	2.0 (0.4–8.8)	58 (9)	45 (12)	1.1 (0.6–1.9)

BMI=body mass index; CI=confidence interval; OR=odds ratio.

a Adjustments were made for age, educational level, alcohol use, gastro-oesophageal reflux, cigarette smoking, intake of fruits and vegetables, and *Helicobacter pylori* infection.

**Table 5:** Cigarette smoking and risk of oesophageal and cardia adenocarcinoma expressed in OR with 95% CI

	**Women**			**Men**		
	**Controls**	**Cases**	**OR** a	**Controls**	**Cases**	**OR** a
**Variables**	***N*=140 (%)**	***N*=63 (%)**	**(95% CI)**	***N*=676 (%)**	***N*=388 (%)**	**(95% CI)**
*Frequency of smoking (cigarettes per day)*						
Never	91 (65)	29 (46)	1.0 (reference)	275 (41)	98 (25)	1.0 (reference)
1–9	18 (13)	14 (22)	3.4 (1.3–9.4)	119 (18)	64 (17)	1.5 (1.0–2.3)
10–19	24 (17)	14 (22)	2.4 (0.9–6.7)	132 (20)	105 (27)	2.4 (1.6–3.5)
>19	7 (5)	6 (10)	5.7 (1.3–25.6)	150 (22)	121 (31)	2.3 (1.6–3.4)
*P* value for trend						
						
*Duration of smoking*						
Never	90 (64)	29 (46)	1.0 (reference)	233 (35)	71 (18)	1.0 (reference)
1–20 years	17 (12)	5 (8)	1.5 (0.4–5.6)	138 (20)	75 (19)	1.7 (1.1–2.7)
21–35 years	16 (11)	14 (22)	3.9 (1.3–11.4)	140 (21)	100 (26)	2.3 (1.5–3.5)
>35 years	17 (12)	15 (24)	3.6 (1.3–10.0)	165 (24)	142 (37)	3.6 (2.4–5.4)
						
*Years since cessation of cigarette smoking*						
Never	91 (65)	29 (46)	1.0 (reference)	275 (41)	98 (25)	1.0 (reference)
0–2	22 (16)	21 (33)	5.2 (1.9–13.8)	129 (19)	106 (27)	2.6 (1.8–3.9)
3–10	7 (5)	5 (8)	3.3 (0.7–14.9)	54 (8)	50 (13)	2.7 (1.7–4.5)
11–25	11 (8)	6 (10)	2.4 (0.6–9.9)	101 (15)	76 (20)	2.0 (1.3–3.1)
>25	9 (6)	2 (3)	0.8 (0.1–4.8)	117 (17)	58 (15)	1.4 (0.9–2.2)
						
*Pack year of cigarette smoking* b						
1	91 (65)	29 (46)	1.0 (reference)	275 (41)	98 (25)	1.0 (reference)
2	21 (15)	10 (16)	2.6 (0.9–7.4)	141 (21)	78 (20)	1.6 (1.1–2.4)
3	13 (9)	13 (21)	4.3 (1.4–13.0)	125 (19)	81 (21)	1.8 (1.2–2.7)
4	15 (11)	11 (17)	3.1 (1.0–9.6)	135 (20)	131 (34)	2.9 (2.0–4.2)

BMI=body mass index; CI=confidence interval; OR=odds ratio.

a Adjustments were made for age, educational level, alcohol use, gastro-oesophageal reflux, BMI, intake of fruits and vegetables, and *Helicobacter pylori* infection.

b Number of cigarettes per day times the total years of smoking.
